# Understanding dysnatremia

**DOI:** 10.1007/s10877-020-00512-z

**Published:** 2020-05-07

**Authors:** Philip J. G. M. Voets, Nils P. J. Vogtländer, Karin A. H. Kaasjager

**Affiliations:** 1 Department of Nephrology, Gelre Hospital, Albert Schweitzerlaan 31, 7334 DZ Apeldoorn, The Netherlands; 2 Department of Nephrology, University Medical Centre Utrecht, Utrecht, The Netherlands

**Keywords:** Dysnatremia, Plasma sodium concentration, Mathematical prediction model

## Abstract

Dysnatremia—either hyponatremia or hypernatremia—is frequently encountered in the clinical practice and often poses a diagnostic and therapeutic challenge for physicians. Despite their frequent occurrence, disorders of the water and sodium balance in the human body have puzzled many physicians over the years and often remain elusive for those lacking experience in their interpretation and management. In this article, we derive a transparent governing equation that can be used by clinicians to describe how a change in relevant physiological parameters will affect the plasma sodium concentration. As opposed to many existing models, our model takes both input and output into account, and integrates osmolarity and tonicity. Our governing equation should be considered a means for clinicians to get a better qualitative understanding of the relationship between the plasma sodium concentration and the variables that influence it for a wide range of scenarios.

## Introduction

Dysnatremia—either hyponatremia or hypernatremia—is frequently encountered in the clinical practice and often poses a diagnostic and therapeutic challenge for physicians [1, 2]. Although many dysnatremic patients remain asymptomatic (especially if a change in the plasma sodium concentration is mild and the onset is gradual), dysnatremia can cause debilitating symptoms, such as nausea, lethargy, and seizures, and has consistently been associated with a higher mortality in hospitalized patients [1, 2]. However, despite their frequent occurrence, disorders of the water and sodium balance in the human body have puzzled many physicians over the years and often remain elusive for those lacking experience in their interpretation and management [1].

In this article, we propose a transparent governing equation [Eq. (11)] that provides insight into how a change in relevant physiological parameters affects the plasma sodium concentration. As opposed to many existing models, such as the renowned Adrogue–Madias equation, our model takes both input and output into account, and integrates osmolarity and tonicity [3–5]. It is important to note that it is not the aim of this equation to calculate changes in the plasma sodium concentration exactly, and it does not remove the need for frequent plasma sodium measurements while treating dysnatremia. Rather, the derived equation should be considered a useful means for clinicians to get a better qualitative understanding of the relationship between the plasma sodium concentration and the physiological variables that influence it. Therefore, an experimental validation of our model falls beyond the scope of this article.

Below, the mathematical derivation of Eq. (11) will be discussed stepwise.

## Mathematical derivation

The plasma sodium concentration (${\left[{\mathrm{N}\mathrm{a}}^{+}\right]}_{p}$) can be fairly accurately described by the simplified Edelman equation as a function of the total body exchangeable sodium and potassium (${{\mathrm{N}\mathrm{a}}_{e}}^{+}+{{\mathrm{K}}_{e}}^{+}$) and the total body water ($TBW$) [6, 7]:

1 $${\left[{\mathrm{N}\mathrm{a}}^{+}\right]}_{p}=\frac{{{\mathrm{N}\mathrm{a}}_{e}}^{+}+{{\mathrm{K}}_{e}}^{+}}{TBW}.$$

It has been shown experimentally that the use of Eq. (1) sometimes leads to a slight—but clinically allowable—overestimation of the plasma sodium concentration [7]. For the purpose of deriving our qualitative model, this small deviation from the original, but mathematically more intricate, Edelman equation was deemed acceptable.

A change in plasma sodium concentration is determined by the change in electrolyte-free total body water ($\Delta TBW$), assuming that the total amount of exchangeable sodium and potassium does not change [6–8]:

2 $$\Delta {\left[{\mathrm{N}\mathrm{a}}^{+}\right]}_{p}=\frac{{{\mathrm{N}\mathrm{a}}_{e}}^{+}+{{\mathrm{K}}_{e}}^{+}}{TBW+\Delta TBW}-\frac{{{\mathrm{N}\mathrm{a}}_{e}}^{+}+{{\mathrm{K}}_{e}}^{+}}{TBW}.$$

We have previously shown that—under the reasonable condition that $TBW\gg \Delta TBW$ holds true—the equation above can be algebraically reduced to [8]:

3 $$\Delta {\left[{\mathrm{N}\mathrm{a}}^{+}\right]}_{p}=-{\left[{\mathrm{N}\mathrm{a}}^{+}\right]}_{p}\frac{\Delta TBW}{TBW}.$$

The net change in electrolyte-free total body water can be described as the difference between the electrolyte-free total body water input ($EFWI$), both oral and parenteral, and the electrolyte-free total body clearance ($EFWC$) [9–11]:

4 $$\Delta TBW=EFWI-EFWC.$$

In contrast to the traditional concept of solute-free water, electrolyte-free water ignores osmotically inert solutes, such as urea. Equation (4) should be considered invalid in the case of significant volume redistributions between the intracellular and extracellular compartment, which primarily occurs in plasma hypertonicity and severe dehydration. In this derivation, the insensible body water losses (such as through perspiration) in the period between plasma sodium concentration measurements are considered negligible.

The equation for electrolyte-free total body water input and the electrolyte-free total body clearance are as follows [8, 9, 11]:

5.1 $$EFWI={V}_{i}\left(1-\frac{{\left[{E}^{+}\right]}_{i}}{{\left[{\mathrm{N}\mathrm{a}}^{+}\right]}_{p}}\right),$$

5.2 $$EFWC={V}_{u}\left(1-\frac{{\left[{E}^{+}\right]}_{u}}{{\left[{\mathrm{N}\mathrm{a}}^{+}\right]}_{p}}\right).$$

In which ${V}_{i}$ and ${V}_{u}$ represent the input flow rate and urine output flow rate, respectively. With regard to the mathematical transparency of our model, effective non-electrolyte solutes (e.g., glucose and mannitol) are not incorporated in the equations above and their effect on tonicity is assumed to be small compared to the effect of electrolytes. Clearly, this assumption is not valid for hypertonic hyponatremia. Substitution of these equations in Eq. (4) produces [8]:

6 $$\Delta TBW={V}_{i}\left(1-\frac{{\left[{E}^{+}\right]}_{i}}{{\left[{\mathrm{N}\mathrm{a}}^{+}\right]}_{p}}\right)-{V}_{u}\left(1-\frac{{\left[{E}^{+}\right]}_{u}}{{\left[{\mathrm{N}\mathrm{a}}^{+}\right]}_{p}}\right).$$

Therefore:

7.1 $$\Delta {\left[{\mathrm{N}\mathrm{a}}^{+}\right]}_{p}=-\frac{{\left[{\mathrm{N}\mathrm{a}}^{+}\right]}_{p}}{TBW}\left({V}_{i}\left(1-\frac{{\left[{E}^{+}\right]}_{i}}{{\left[{\mathrm{N}\mathrm{a}}^{+}\right]}_{p}}\right)-{V}_{u}\left(1-\frac{{\left[{E}^{+}\right]}_{u}}{{\left[{\mathrm{N}\mathrm{a}}^{+}\right]}_{p}}\right)\right),$$

7.2 $$\Delta {\left[{\mathrm{N}\mathrm{a}}^{+}\right]}_{p}=\frac{{V}_{u}\left({\left[{\mathrm{N}\mathrm{a}}^{+}\right]}_{p}-{\left[{E}^{+}\right]}_{u}\right)-{V}_{i}\left({\left[{\mathrm{N}\mathrm{a}}^{+}\right]}_{p}-{\left[{E}^{+}\right]}_{i}\right)}{TBW}.$$

The numerator and denominator in Eq. (7.2) are multiplied by 2, which produces:

8 $$\Delta {\left[{\mathrm{N}\mathrm{a}}^{+}\right]}_{p}=\frac{2{V}_{u}\left({\left[{\mathrm{N}\mathrm{a}}^{+}\right]}_{p}-{\left[{E}^{+}\right]}_{u}\right)-{2V}_{i}\left({\left[{\mathrm{N}\mathrm{a}}^{+}\right]}_{p}-{\left[{E}^{+}\right]}_{i}\right)}{2TBW}.$$

The terms $2\left({\left[{\mathrm{N}\mathrm{a}}^{+}\right]}_{p}-{\left[{E}^{+}\right]}_{u}\right)$ and $2\left({\left[{\mathrm{N}\mathrm{a}}^{+}\right]}_{p}-{\left[{E}^{+}\right]}_{i}\right)$—in which the factor 2 accounts for the anions—can broadly be redefined as the difference in tonicity between the plasma and urine ($\Delta {T}_{p,u}$) and the difference in tonicity between plasma and input ($\Delta {T}_{p,i}$), respectively:

9.1. $$\Delta {T}_{p,u}=2\left({\left[{\mathrm{N}\mathrm{a}}^{+}\right]}_{p}-{\left[{E}^{+}\right]}_{u}\right),$$

9.2 $$\Delta {T}_{p,i}=2\left({\left[{\mathrm{N}\mathrm{a}}^{+}\right]}_{p}-{\left[{E}^{+}\right]}_{i}\right).$$

Equations (9.1) and (9.2) can be substituted in Eq. (10), which produces:

10 $$\Delta {\left[{\mathrm{N}\mathrm{a}}^{+}\right]}_{p}=\frac{{\Delta {T}_{p,u}V}_{u}-{\Delta {T}_{p,i}V}_{i}}{2TBW}.$$

Because two times the total body water approximately equals body weight ($W$), and because the obligatory urine production is determined by the ratio of osmoles that need to be excreted ($N$) to osmole excretion per liter of urine (i.e., the urine osmolarity or ${O}_{u}$), Eq. (10) can be rewritten to [12, 13]:

11 $$\Delta {\left[{\mathrm{N}\mathrm{a}}^{+}\right]}_{p}=\frac{\Delta {T}_{p,u}N/{O}_{u}-\Delta {T}_{p,i}{V}_{i}}{W}.$$

Although the simultaneous use of urine osmolarity and tonicity in the equation above may seem inconsistent at first, it is important to note that, while the electrolyte-free water balance ultimately determines the change in the plasma sodium concentration, the urine flow rate itself—which sets a limit on the amount of electrolyte-free water loss—is determined by the rate of solute excretion (which includes inert solutes, such as urea) [13].

## Discussion

In the previous section, a governing dysnatremia equation has been derived that describes the effect of a change in any of the physiological parameters on the change in the plasma sodium concentration. Our model can be applied to a wide range of clinical dysnatremia scenarios, several of which will be discussed below.

It is well-known that the osmole intake of a person strongly influences his or her water and sodium balance [12, 13]. Therefore, changes in osmole intake frequently cause, or predispose for, dysnatremia. Equation (11) clearly demonstrated that a significant decrease in osmole intake—which is reflected by a decreased value for $N$, as fewer osmoles need to be excreted—predisposes for drop in plasma sodium concentration [12–15]. Among clinicians, this is also known as ‘tea and toast syndrome’, and it is often encountered in the elderly and the malnourished [13, 14]. As a compensatory mechanism, the kidneys will optimize their renal water excretion by minimizing the osmole excretion per liter of urine, which is reflected by a decrease in ${O}_{u}$, correcting the aforementioned ratio $N/{O}_{u}$ [12–15]. Because the urine cannot be composed of pure water, but must contain a minimum amount of osmoles (approximately 100 mOsmol/L), this compensatory mechanism will eventually fail when the urine cannot be diluted any further while a patient continues to ingest a significant volume of hypotonic fluids, such as beer or even pure water [13]. The inability of the human body to get rid of the introduced water load due to a lack of osmoles that can be excreted in order to produce urine, leads to a water excess and hypotonic hyponatremia [13–15]. Administering normal saline to these patients will increase their plasma sodium concentration much more than would be expected from the simple redistribution of the introduced infusate, which can be calculated by the Adrogue–Madias equation [3–5]. Unlike the Adrogue–Madias equation, our model shows that both the reintroduction of solutes—reflected by an increased value for $N$—which greatly enhances urinary water excretion in these patients, and the relative hypertonicity of normal saline compared to their hypotonic hyponatremic plasma (and thus a negative value for $\Delta {T}_{p,i}$) contribute to this increase in their plasma sodium concentration. Analogously, as the osmole input and therefore the value for $N$ strongly increases (e.g., in parenterally fed patients), hyperalimentation hypernatremia can develop due to an increase in urinary water loss [15].

With regard to differences in input and output tonicity, it stands to reason that the plasma sodium concentration will drop as the value for $\Delta {T}_{p,u}$ becomes smaller, and the value for $\Delta {T}_{p,i}$ becomes larger. This reflects a situation in which the urine becomes hypertonic, whereas the input consists of more hypotonic fluids. An example of the latter is primary polydipsia, which would result in an increased value for both ${V}_{i}$ (due to the large volume of ingested fluids) and $\Delta {T}_{p,i}$ (due to the low electrolyte concentration in the ingested fluids and thus the low value for ${\left[{E}^{+}\right]}_{i}$, resulting in an increase in the value for $2\left({\left[{\mathrm{N}\mathrm{a}}^{+}\right]}_{p}-{\left[{E}^{+}\right]}_{i}\right)$) [13–15]. Conversely, any increase in $\Delta {T}_{p,u}$ (i.e., by reducing the urinary electrolyte excretion, which lowers ${\left[{E}^{+}\right]}_{u}$ and thus increases the value for $2\left({\left[{Na}^{+}\right]}_{p}-{\left[{E}^{+}\right]}_{u}\right)$) and/or decrease in $\Delta {T}_{p,i}$ will predispose for a rise in the plasma sodium concentration [14].

In the clinical practice, hypotonic hyponatremia is often the result of excessive production of antidiuretic hormone (ADH), which stimulates pure water retention in the collecting ducts [16, 17]. Under physiological conditions, the plasma osmolarity determines the degree of ADH release from the pituitary gland. However, in the case of intravascular volume depletion (which often occurs as a result of the chronic use of diuretics, diarrhea, vomiting, adrenal insufficiency or forward failure due to cardiac pathology), a hypovolemic stimulus can override coexisting osmotic stimuli and trigger the release of ADH [17, 18]. In Eq. (11), this increases the value for ${O}_{u}$. The effect of hypovolemia on $\Delta {T}_{p,u}$ is more difficult to predict, as this parameter is strongly influenced by the degree of natriuresis and the urine flow rate, and therefore depends on the specific cause of hypovolemia [13]. Regardless of the underlying cause, removing the hypovolemic stimulus for ADH release by treating the underlying pathology and/or by initiating intravenous fluid therapy promotes renal water excretion, reduces ${O}_{u}$, and often corrects the hypotonic hyponatremia [17, 18]. Another frequently encountered example of excessive ADH release is the syndrome of inappropriate antidiuretic hormone secretion (SIADH), which is frequently caused by lung disease, medication, malignancy or disorders of the central nervous system [16, 17]. According to a classical clinical dogma, normal saline should be avoided in SIADH patients with a high urine osmolarity, as the kidneys were believed to simply excrete the introduced electrolytes, while retaining the introduced water. However, Eq. (11) shows that SIADH patients can be effectively treated with normal saline, even in the setting of a relatively high urine osmolarity as long as the urine tonicity remains sufficiently low (i.e., the value for $\Delta {T}_{p,u}$ remains relatively high) [8, 16, 18]. This corresponds with clinical observations by—among others—Shimizu et al., Hoorn et al. and Zietse et al. [10, 18, 19]. Administering normal saline to SIADH patients with a high urine tonicity due to significant natriuresis (which is further amplified by administering saline infusate) and therefore a negative value for $\Delta {T}_{p,u}$ (i.e., ${\left[{E}^{+}\right]}_{u}>{\left[{\mathrm{N}\mathrm{a}}^{+}\right]}_{p}$) will most likely exacerbate their initial hypotonic hyponatremia, whereas SIADH patients with a relatively low urine tonicity and therefore a positive value for $\Delta {T}_{p,u}$ may benefit from saline infusion, regardless of their urine osmolarity [8, 15–17]. In diabetes insipidus, which is in many ways the opposite of SIADH, massive urinary water loss dilutes urinary electrolytes and increases the value for $\Delta {T}_{p,u}$, which results in hypernatremia [15–17]. By drinking sufficient amounts of electrolyte-free water (with a high value for ${V}_{i}$ and ${\left[{E}^{+}\right]}_{i}=0$), and by taking diuretics such as amiloride (which reduce the value for $\Delta {T}_{p,u}$), the plasma sodium concentration can be decreased effectively [17].

As described above, Eq. (11) can be used to describe the plasma sodium response—and the renal compensatory response—for a wide range of scenarios. The magnitude of the aforementioned changes in the plasma sodium concentration will, in part, depend on the initial amount of total body water [17, 20]. The total body water is proportional to body mass (represented by the term $W$); i.e., the larger the body weight, the smaller the impact of a parameter change on the plasma sodium concentration, and vice versa [17, 20]. Our mathematical model can also be applied to interpret complex cases of multifactorial dysnatremia, in which multiple factors simultaneously—but not necessarily synergistically—contribute to an observed change in the plasma sodium concentration. However, Eq. (11) should not be applied to cases of hypertonic hyponatremia (such as overt hyperglycemia), as the effect of effective non-electrolyte solutes on the input and output tonicity balance is considered relatively insignificant. These solutes are thus ignored in the presented tonicity balance, as we feel that incorporating these solutes in the Eqs. (5.1) and (5.2) would greatly diminish the mathematical transparency and clinical utility of our final equation [21].

As a concluding remark, it stands to reason that patient characteristics should be considered in the analysis of every disorder of the water and sodium balance and that frequent measurements of the plasma sodium concentration remain imperative. We would like to emphasize again that the presented model is a transparent tool for the analysis of dysnatremia, which is not intended for exact calculations.

## Abbreviations

ADH: Antidiuretic hormone
SIADH: Syndrome of inappropriate antidiuretic hormone secretion
${\left[{\mathrm{N}\mathrm{a}}^{+}\right]}_{p}$: Plasma sodium concentration
$\Delta {\left[{\mathrm{N}\mathrm{a}}^{+}\right]}_{p}$: Change in plasma sodium concentration
${{\mathrm{N}\mathrm{a}}_{e}}^{+}+{{\mathrm{K}}_{e}}^{+}$: Total body exchangeable sodium and potassium
$\Delta {T}_{p,u}$: Plasma tonicity minus urine tonicity
$\Delta {T}_{p,i}$: Plasma tonicity minus input tonicity
${\left[{E}^{+}\right]}_{i}$: Cation concentration of input
${\left[{E}^{+}\right]}_{u}$: Cation concentration of urine
$${O}_{u}$$: Urine osmolarity
${V}_{u}$: Urine output flow rate
${V}_{i}$: Input flow rate
$EFW$: Electrolyte-free total body water
$EFWI$: Electrolyte-free total body water input
$EFWC$: Electrolyte-free total body water clearance
$TBW$: Total body water (0.6 times body weight for men, 0.5 times body weight for women)
$\Delta TBW$: Change in electrolyte-free total body water
$N$: Obligatory osmole excretion
